# Differences in Fat Mass Estimation Formulas in Physically Active Adult Population and Relationship with Sums of Skinfolds

**DOI:** 10.3390/ijerph17217777

**Published:** 2020-10-23

**Authors:** Raquel Vaquero-Cristóbal, Mario Albaladejo-Saura, Ana E. Luna-Badachi, Francisco Esparza-Ros

**Affiliations:** 1 Faculty of Sport, Catholic University San Antonio of Murcia (UCAM), Av. de los Jerónimos 135, 30107 Murcia, Spain; rvaquero@ucam.edu; 2 Kinanthropometry International Chair, Catholic University San Antonio of Murcia (UCAM), Av. de los Jerónimos 135, 30107 Murcia, Spain; mdalbaladejosaura@ucam.edu (M.A.-S.); anaelenalunab@gmail.com (A.E.L.-B.)

**Keywords:** kinanthropometry, physical performance, body weight, health

## Abstract

Changes in body composition and specifically fat mass, has traditionally been used as a way to monitor the changes produced by nutrition and training. The objective of the present study was to analyse the differences between the formulas used to estimate fat mass and to establish the existing relationship with the body mass index and sums of skinfolds measurement in kinanthropometry. A total of 2458 active adults participated in the study. Body mass index (BMI) and skinfolds were measured, and the Kerr, Durnin-Womersley, Faulkner and Carter equations were used to assess fat mass. Significant differences were found between all the formulas for the percentage of fat mass, ranging from 10.70 ± 2.48 to 28.43 ± 5.99% (*p* < 0.001) and fat mass from 7.56 ± 2.13 to 19.89 ± 4.24 kg (*p* < 0.001). The correlations among sums of skinfolds and the different equations were positive, high and significant in all the cases (*r* from 0.705 to 0.926 *p* < 0.001), unlike in the case of BMI, were the correlation was lower and both positive or negative (*r* from −0.271 to 0.719; *p* < 0.001). In conclusion, there were differences between all the formulas used to estimate fat mass; thus, for the evaluation of fat mass with kinanthropometry of an active adult, the use of the same formula is recommended on all occasions when the results are going to be compared or when an athlete is compared with a reference.

## 1. Introduction

The changes in body composition, and specifically the percentage of fat mass, have traditionally been used as a way to monitor the effects produced by training and nutrition in athletes and non-athlete population [1,2,3,4]. Nutritional interventions and training programs aimed at improving the performance of athletes have also been shown to be effective in reducing fat mass [5,6]. In fact, there is a relationship between fat mass and sports performance [7,8], because an excess of fat mass reduces sports performance in most sport modalities such as aesthetic and endurance sports [9,10]. Athletes can be characterized according to the type of sport and the specific field position according to fat mass [11].

However, there is also a relationship between fat mass and health indicators of different groups of non-athlete populations [12]. Fat mass can play an important role in the current obesity epidemic, which has become a major public health problem [13,14]. In fact, obesity is defined as the accumulation of excess body fat to the extent that it may have adverse effects on health [15]. Furthermore, excessive fat mass is associated with other health risk factors such as elevated plasma cholesterol, plasma glucose, and resting blood pressure, which contribute to the development of cardiovascular disease and type 2 diabetes, among other diseases [14,16,17,18].

In the scientific literature, a multitude of methods for assessing body composition can be found [19,20]. The most common methods for performing an in vivo assessment of body composition are bioimpedance (BIA), dual-energy X-ray absorptiometry (DXA) and kinanthropometry, among others, and all of them have positive and negative aspects [21]. BIA is a method based on the electrical conductivity properties of the human body, and its main advantages are that it is a safe, observer-independent, inexpensive field test and it is easy to perform [14]. However, some limitations have been showed by this method, such as its low sensitivity in the evaluation of the trunk; the influence of previous exercise, body position, skin temperature or dietary intake; the need of the examined subject to match the reference population from which the regression equation was obtained, to utilize a valid method because conductivity depends on age, ethnicity, hydration, health status, etc.; or a controversial assessment of longitudinal changes in fat mass when significant weight loss occurs due to concurrent changes in volume and composition of the conducting tissue. In addition, the portability of the most valid models is limited [14,22,23,24,25].

DXA is based on the attenuation of calibrated X-ray beams with dual photon energy so each atomic element has a characteristic mass attenuation coefficients for a given photon energy [14]. The advantages of DXA as a method of body composition measurement include observer independence, excellent precision for whole-body measurements, modest demands for the cooperation of the patient [14]. However, there are some negative aspects in the body composition evaluation with this method as well. For example, the equipment is expensive, the influence of the inaccurate positioning of the subjects on the final result; presence of metal implants; administration of radioactive substances to the subject; bias in determination of body fat from 38% to 28%; underestimation of fat mass in lean individuals; a variability from 0.8% to 27% in sequential measurements; or that it is not a portable method [14,26].

Alternatively, kinanthropometry uses the measurement of certain skinfolds to establish a percentage of fat mass through the use of different equations [19,20]. The use of skinfold thickness to estimate body fat is based on the relationship between subcutaneous adipose tissue and total body fat [14]. Its principal advantages are that it is easy, inexpensive, easy to transport and quick to apply [19,27]. Furthermore, kinanthropometry has been shown to have sufficient validity and reliability to be considered a useful tool in the assessment of fat percentage in the athlete population [28]. However, reliability can be affected by the error introduced by the researcher [29]. In fact, the error introduced by a non-expert researcher can vary between 3% and 11% [19,20,29].

If all the methods to estimate body composition have advantages and defects, there is a clear trend among professionals interested in body composition to choose methods with a better benefit to cost ratio, and the kinanthropometric method as a field test is one of the most widely used in the assessment of body composition of athletes [20]. However, the despite different methods for assessing body composition demonstrating reliability and validity in samples of the general population and athletes, it has been shown that it is not possible to compare between the body composition results obtained with different methods [28,30]. Furthermore, many formulas have been validated to estimate fat mass through kinanthropometry [29], and no studies have been found that compare the variety of fat masses depending on the formula used to estimate it with kinanthropometry. This comparison is needed in order to verify if references and measurements with different formulas can be compared, even when these formulas have been validated in similar populations. In addition, it is common to find that when fat mass is estimated in athletes with kinanthropometry, most of the attention is placed on the result (percentage or kg), without any importance attached to the formula chosen for the calculation, despite the importance it could have on the final result.

Therefore, the objective of the present study was to analyze the differences between the formulas used to estimate fat mass with kinanthropometry and to establish the existing relationship with the body mass index and sums of skinfolds in kinanthropometry. 

## 2. Materials and Methods 

### 2.1. Participants

The calculations for establishing the sample size were performed using Rstudio 3.15.0 software (Rstudio Inc., Boston, MA, USA). The significance level was set at α = 0.05. The standard deviation (SD) was established based on previous studies of fat mass (mean SD = 1.7) [10], the percentage of fat mass (mean SD = 2.3), sum of six skinfolds (mean SD = 8.4) and sum of eight skinfolds in kinanthropometry (mean SD = 9.7) [31]. With an estimated error (d) of 0.067 kg of fat mass, of 0.092% in the percentage of fat mass, 0.33 mm in the sum of six skinfolds and 0.38 mm in the sum of eight skinfolds in kinanthropometry. The sample needed was 2458 subjects.

Participants were volunteers from the Region of Murcia (Spain) and the selection of the participants was non-probabilistic for convenience. The inclusion criteria were (1) to be aged between 18 and 50 years old and (2) to practice any sport or exercise on a regular basis. To determine if the participants could be considered physically active, the IPAQ questionnaire was utilized [32], and the Global Recommendations on Physical Activity for Health of the World Health Organization were set as the criteria [33].

### 2.2. Procedure

The Institutional Ethical Committee reviewed and authorized the protocol designed for data collection according to the Code of the World Medical Association (number 23/02/2013). The statements of the Declaration of Helsinki were followed during the entire process. All the participants were informed about the procedures and signed an informed consent form before the start of the study. The protocol was registered (ClinicalTrials.gov Identifier: NCT04429581).

Kinanthropometric variables were measured following the guidelines of the International Society for the Advancement in Kinanthropometry (ISAK) [34] between 2013 and 2018. Basic measurements of body mass and stretch stature; and the triceps, subscapular, biceps, iliac crest, supraspinale, abdominal, thigh and calf skinfolds were measured by level 2, 3 and 4 anthropometrists accredited by the ISAK. The intra- and inter-evaluator technical error of measurement (TEM) were calculated in a sub-sample. The intra-evaluator TEM was 0.01% in basic measures and 1.13% in skinfolds; and the inter-evaluator TEM was 0.03% in basic measures and 2.97% in skinfolds.

A SECA 862 scale (SECA, Hambourg, Germany) with 100 g precision was used to measure body mass; a SECA stadiometer (SECA, Hambourg, Germany) with 0.1 mm precision was used for the assessment of stretch stature, while skinfolds were measured with a Harpenden caliper (Harpenden, London, UK), with a precision of 0.2 mm.

Each measurement was performed twice. In case the differences between measurements was higher than 1% in basic measurements or 5% in skinfolds, the measurement was performed a third time. The final value for the data analysis was the mean if two measurement were taken or the median if three measurements were taken. The participants were told to avoid heavy foods and physical activity starting from the day before the measurement session. All the measurements were performed in a room with a standardized temperature of 24 °C, from 9:00 to 14:00. 

The BMI, sum of six skinfolds (triceps, subscapular, supraespinale, abdominal, thigh and calf) and sum of eight skinfolds (triceps, subscapular, biceps, iliac crest, supraspinale, abdominal, thigh and calf) were calculated. The equations used to estimate fat mass and the percentage of fat mass were those proposed by Kerr [35], Durning-Womersley [7], Faulkner [36] and Carter [37]. All of them had been validated in the measurement of athletes.

### 2.3. Statistical Analysis

The normality of the distribution was checked with the Kolmogorov-Smirnov test. The kurtosis analysis showed a platikurtic distribution for all the variables. All the variables included in the analysis followed a normal distribution, so a parametric statistics test was performed. A descriptive statistics test was performed for all the variables. The differences between fat mass equations were analysed with a one-way analysis of variance (ANOVA) for repeated measurements. The confidence interval (CI) of the differences (CI of 95%) was included and effect size (Cohen´s D) was calculated [38]. Threshold values for effect size were set as ≥ 0.2 (small), ≥ 0.5 (moderate) and ≥ 0.8 (large). Pearson’s correlation was performed between BMI, sums of six and eight skinfolds and fat mass equations. Percentiles relating all the variables were included in the analysis. The level of significance was set at *p* ≤ 0.05. The software used to perform the statistical analysis was SPSS (v.23, IBM, Endicott, NY, USA). 

## 3. Results

The present study was conducted with a sample of 2458 active subjects (mean age: 27.98 ± 7.43 years-old; mean metabolic equivalents (MET)—minute/week: 3728.63 ± 132,815), of which 1775 were men (mean age: 27.99 ± 7.46 years) and 681 was women (mean age: 27.94 ± 7.37 years). The descriptive analysis of body mass index (BMI), sums of skinfolds and fat masses and percentages estimated are shown in Table 1.

### 3.1. Differences Between Fat Mass Prediction Equations

The analysis of the differences between fat mass equations can be observed in Table 2 and Table 3. All the equations used to estimate fat mass and the percentage of fat mass showed statistical differences (*p* < 0.001). The effect side observed was high in all the cases (D from 0.87 to 3.86) (Table 2 and Table 3).

### 3.2. Correlations Among Fat Mass Equations, Sum of Skinfolds and BMI

The Pearson’s correlation analysis between BMI, sums of skinfolds and fat masses and fat percentages are shown in Table 4 and Table 5, respectively. The correlations of the different equations were positive, high and significant between themselves (*r* from 0.732 to 0.940; *p* < 0.001). The correlations between BMI and six and eight sums of skinfolds was significant, but low in both cases (*r* = 0.187 and 0.218, respectively; *p* < 0.001) (Table 4 and Table 5).

A positive low to high positive correlation was found between BMI and most of the fat mass and percentage equations (*r* from 0.187 to 0.719; *p* < 0.001); although a negative low correlation was found with fat percentages estimated by Kerr and Durnin-Womersley (*r* = −0.271 and −0.055, respectively; *p* < 0.001). A positive moderate to high correlation was found among fat mass equations and the sums of six and eight skinfolds (*r* from 0.613 to 0.849; *p* < 0.001). 

The percentile analysis relating the measurements included in the study can be observed in Table 6.

## 4. Discussion

The objective of the present study was to analyze the differences between the formulas used to estimate fat mass with kinanthropometry and to establish the existing relationship with sums of skinfolds. It has been observed that even when using the same method to estimate fat mass, problems can occur when comparing results. In widely-used methods for body estimation, such as BIA, it has been observed that various factors can produce inaccuracies between measurements [39]. Among these factors, those related to the placement of the electrodes and the equations used stand out, making the results obtained vary by around 4% between instruments [39]. The same occurs with DXA, and significant differences in body composition have been shown when comparing machines from different manufacturers, different models from the same manufacturer or the same scanner model [40,41,42,43], or the version of the software. However, previous studies focused on the use of kinanthropometry have not been found. The main finding was that there are significant differences between all the formulas. In fact, differences found between the fat mass formulas ranged from 10.70 ± 2.48% to 28.43 ± 5.99 % and from 7.56 ± 2.13 kg to 19.89 ± 4.24 kg although all the formulas had been validated with active adults [7,35,36,37]. These data agree with what was found in the previous literature, supporting the idea that there are significant intra-method differences when using various formulas to estimate fat mass [44,45].

The differences found may be due to the method used in the validation of the formula, as Kerr used cadaver dissection as a method to compare the results [35], while Durnin-Womersley, Faulkner and Carter used hydrostatic weighing [7,36,37]. The hydrostatic weighing assumes certain sources of error. The body density is obtained by weighing the subject in air and fully submerged [20]. The air found in the lungs and in the gastrointestinal (GI) tract is one of the factors that can affect body density [20]. There is a consensus about the value given to the GI tract air, set as 100 mL [46], but the residual volume of the lungs generates uncertainty [47], perhaps affecting the final result. Once the body density value is obtained, estimation equations are used to estimate the fat percentage [20]. These equations use a constant value for the density of fat mass and free fat mass [20]. While the use of a constant value for fat mass is well accepted, free fat mass has been demonstrated to be affected by age, gender and race [48,49,50], thereby being another aspect of bias. Thus, Kerr [35] is the only formula that was validated with a direct method, while the other formulas were validated with indirect methods. Therefore, an error in the estimation of fat mass with these methods has to be assumed [29]. 

Another reason why these differences may occur is due to the diversity of the target population [45]. Although all of participants were adults, the Kerr [35] and Durnin-Womersley [7] formulas were validated in the general population. Instead, the equations proposed by Faulkner and Carter were validated in elite athlete populations, specifically in swimmers and Olympic athletes, respectively [36,37]. Other factors that may introduce differences in the calculation of body composition are gender and age [39]. In this way, Kerr’s proposed a unique fat mass prediction formula [35]; Durnin-Womersley proposed different equations according to the age group [7]; while Faulkner and Carter established a formula for men and another for women [36,37]. 

However, statistically significant and positive correlations were observed between all fat mass formulas (*r* from 0.705 to 0.926; *p* < 0.001). This can be attributed to all of these formulas using skinfolds, as Carter and Kerr proposed the use of the sum of six skinfolds [35,37], Durnin-Womersley used four skinfolds (triceps, biceps, subscapular and iliac crest) [7] and Faulkner used another four skinfolds (triceps, subscapular, supraspinale and abdominal) [36]. In fact, a high correlation was found between fat mass formulas with sums of six and eight skinfolds (*r* from 0.613 to 0.849; *p* < 0.001). The skinfolds represent the subcutaneous fat, 40%-60% of the total body fat [29,51]. Due to the lack of uniformity in the distribution of the thickness of the subcutaneous fat tissue, skinfolds in different locations of the body have to be measured, including trunk, upper and lower limbs, to ensure the correct assessment of fat mass [29,51]. The skinfold measurements from different locations used in the equations analysed could explain the high correlation found between them and the sums of six and eight skinfolds. 

Furthermore, in this study, a low correlation was also observed between BMI and sums of skinfolds and the formulas for estimating fat mass (*r* from 0.055 to 0.338; *p* < 0.001). Despite BMI being a variable that has been traditionally used for weight control, and related with health [52], in an athlete population, it has been shown not to have a sufficient precision to assess the adiposity of the subjects [53]. This may be due to the fact that BMI does not discriminate between the contribution of fatty tissue and muscle tissue to the total weight [54]. The practice of sports can produce lean muscle mass gain [55], and as the muscle is denser than the fat tissue, BMI could not be a valid instrument to assess changes in a physically-active population [20,56]. Furthermore, systematic exercise practice can reduce the sums of skinfolds, as a consequence of the loss of subcutaneous fat, with no changes in BMI because the decrease of fat mass is compensated by the increase in muscle mass [57], which can explain why the correlation between BMI and sums of skinfolds in the present study was also low. 

For all the aforementioned reasons, the same method should always be used to assess fat mass using kinanthropometry and estimation formulas, to reduce the bias introduced by the formula. In fact, fat mass estimation with different formulas cannot be compared, so it is necessary to have a percentile table as it is included in the current study to know the equivalences between methods. However, to avoid this kind of challenges, the sums of six and eight skinfolds are proposed as the measurement of the changes of subcutaneous fat. If the measurements are taken by an experienced researcher and with a calibrated calliper, the error can be reduced with respect to the use of formulas [19,20,29].

Some limitations should be acknowledged. The main limitation was that the assessment of fat mass did not include a comparison between a gold standard and the estimation formulas, so this study is not able to check on the most accurate formula. A comparison with a reference method (e.g., DXA) would have been useful in order to determine the accuracy of each equation. This is an important issue for future research. Other research designs that could be utilized after this study include longitudinal studies to analyse the reliability of body composition measurement with formulas and skinfold sums to find the most appropriate measurement for monitoring changes in body composition.

## 5. Conclusions

There were differences between all the formulas used to estimate fat mass with kinanthropometry. Thus, for the evaluation of fat mass of active adults, the use of the same formula is recommended on all occasions when the results are going to be compared, both to analyse the changes in the athlete or because the athlete’s results are going to be compared with a reference. Furthermore, sums of skinfolds are related with fat mass, thus this method could be an alternative option when a comparison with the same formula is not possible. However, BMI seems not to be a reliable indicator of fat mass in a physically-active population.

## Figures and Tables

**Table 1:** Descriptive analysis of kinanthropometry variables and fat masses and percentages.

Variable	Mean ± SD	Max.	Min.
Height (cm)	173.52 ± 8.58	200.00	148.00
Weight (kg)	70.74 ± 10.89	108.45	43.40
BMI	23.38 ± 2.28	29.98	18.50
Sum of 6 skinfolds (mm)	77.32 ± 23.67	144.80	37.30
Sum of 8 skinfolds(mm)	98.02 ± 29.68	171.00	49.60
FM Kerr (kg)	19.89 ± 4.24	36.37	10.28
FM Durnin-Womersley (kg)	13.55 ± 3.87	28.37	4.96
FM Faulkner (kg)	9.56 ± 2.47	21.08	4.40
FM Carter (kg)	7.56 ± 2.13	15.63	3.18
% Kerr	28.43 ± 5.99	49.49	15.79
% Durnin-Womersley	19.43 ± 5.81	35.30	7.37
% Faulkner	13.46 ± 2.36	22.31	8.98
% Carter	10.70 ± 2.48	17.79	6.50

BMI: Body mass index; FM: fat mass weight; %: percentage of fat mass.

**Table 2:** Differences between fat mass equations in kg.

	FM Kerr	FM Durnin-Womersley	FM Faulkner	FM Carter
FM Kerr	-	Mean differences ± SD:	Mean differences ± SD:	Mean differences ± SD:
		6.33 ± 0.05	10.32 ± 0.05	12.32 ± 0.05
		95% CI: 6.20–6.48	95% CI: 10.19–10.46	95% CI: 12.20–12.44
		*p* < 0.001	*p* < 0.001	*p* < 0.001
		Cohen’s D: 1.56	Cohen’s D: 2.97	Cohen’s D: 3.67
FM Durnin-Womersley	Mean differences ± SD:	-	Mean differences ± SD:	Mean differences ± SD:
	6.33 ± 0.05		3.98 ± 0.05	5.98 ± 0.05
	95% CI: 6.20–6.48		95% CI: 3.84–4.12	95% CI: 5.85–6.11
	*p* < 0.001		*p* < 0.001	*p* < 0.001
	Cohen’s D: 1.56		Cohen’s D: 1.23	Cohen’s D: 1.91
FM Faulkner	Mean differences ± SD:	Mean differences ± SD:	-	Mean differences ± SD:
	10.32 ± 0.05	3.98 ± 0.05		1.99 ± 0.02
	95% CI: 10.19–10.46	95% CI: 3.84–4.12		95% CI: 1.95–2.04
	*p* < 0.001	*p* < 0.001		*p* < 0.001
	Cohen’s D: 2.97	Cohen’s D: 1.23		Cohen’s D: 0.87
FM Carter	Mean differences ± SD:	Mean differences ± SD:	Mean differences ± SD:	-
	12.32 ± 0.05	5.98 ± 0.05	1.99 ± 0.02	
	95% CI: 12.20–12.44	95% CI: 5.85–6.11	95% CI: 1.95–2.04	
	*p* < 0.001	*p* < 0.001	*p* < 0.001	
	Cohen’s D: 3.67	Cohen’s D: 1.91	Cohen’s D: 0.87	

FM: fat mass weight.

**Table 3:** Differences between fat mass equations in percentages.

	% Kerr	% Durnin-Womersley	% Faulkner	% Carter
% Kerr	-	Mean differences ± SD:	Mean differences ± SD:	Mean differences ± SD:
		8.99 ± 0.07	14.97 ± 0.09	17.72 ± 0.08
		95% CI: 8.80–9.19	95% CI: 14.72–15.21	95% CI: 17.51–17.93
		*p* < 0.001	*p* < 0.001	*p* < 0.001
		Cohen’s D: 1.52	Cohen’s D: 3.28	Cohen’s D: 3.86
% Durnin-Womersley	Mean differences ± SD:	-	Mean differences ± SD:	Mean differences ± SD:
	8.99 ± 0.07		5.97 ± 0.09	8.72 ± 0.08
	95% CI: 8.80–9.19		95% CI: 5.73–6.21	95% CI: 8.51–8.84
	*p* < 0.001		*p* < 0.001	*p* < 0.001
	Cohen’s D: 1.52		Cohen’s D: 1.35	Cohen’s D: 1.95
% Faulkner	Mean differences ± SD:	Mean differences ± SD:	-	Mean differences ± SD:
	14.97 ± 0.09	5.97 ± 0.09		2.76 ± 0.02
	95% CI: 14.72–15.21	95% CI: 5.73–6.21		95% CI: 2.70–2.81
	*p* < 0.001	*p* < 0.001		*p* < 0.001
	Cohen’s D: 3.28	Cohen’s D: 1.35		Cohen’s D: 1.14
% Carter	Mean differences ± SD:	Mean differences ± SD:	Mean differences ± SD:	-
	17.72 ± 0.08	8.72 ± 0.08	2.76 ± 0.02	
	95% CI: 17.51-17.93	95% CI: 8.51–8.84	95% CI: 2.70–2.81	
	*p* < 0.001	*p* < 0.001	*p* < 0.001	
	Cohen’s D: 3.86	Cohen’s D: 1.95	Cohen’s D: 1.14	

%: percentage of fat mass.

**Table 4:** Correlation between BMI; sums of skinfolds and fat mass.

	Sum of 6 Skinfolds	Sum of 8 Skinfolds	FM Kerr	FM Durnin-Womersley	FM Faulkner	FM Carter
**BMI**	*r* = 0.187 *p* < 0.001	*r* = 0.218 *p* < 0.001	*r* = 0.331 *p* < 0.001	*r* = 0.411 *p* < 0.001	*r* = 0.719 *p* < 0.001	*r* = 0.613 *p* < 0.001
**Sum of 6 skinfolds**	-	*r* = 0.989 *p* < 0.001	*r* = 0.841 *p* < 0.001	*r* = 0.816 *p* < 0.001	*r* = 0.613 *p* < 0.001	*r* = 0.806 *p* < 0.001
**Sum of 8 skinfolds**		-	*r* = 0.847 *p* < 0.001	*r* = 0.849 *p* < 0.001	*r* = 0.645 *p* < 0.001	*r* = 0.820 *p* < 0.001
**FM Kerr**			-	*r* = 0.793 *p* < 0.001	*r* = 0.840 *p* < 0.001	*r* = 0.939 *p* < 0.001
**FM Durnin-Womersley**				-	*r* = 0.732 *p* < 0.001	*r* = 0.824 *p* < 0.001
**FM Faulkner**					-	*r* = 0.940 *p* < 0.001

**Table 5:** Correlation between BMI, sums of skinfolds and fat mass percentage.

	Sum of 6 Skinfolds	Sum of 8 Skinfolds	% Kerr	% Durnin-Womersley	% Faulkner	% Carter
**BMI**	*r* = 0.187	*r* = 0.218	*r* = −0.271	*r* = −0.055	*r* = 0.338	*r* = 0.187
	*p* < 0.001	*p* < 0.001	*p* < 0.001	*p* < 0.001	*p* < 0.001	*p* < 0.001
**Sum of 6 skinfolds**	-	*r* = 0.989	*r* = 0.889	*r* = 0.812	*r* = 0.916	*r* = 0.999
		*p* < 0.001	*p* < 0.001	*p* < 0.001	*p* < 0.001	*p* < 0.001
**Sum of 8 skinfolds**		-	*r* = 0.865	*r* = 0.821	*r* = 0.929	*r* = 0.989 *p* < 0.001
			*p* < 0.001	*p* < 0.001	*p* < 0.001	
**% Kerr**			-	*r* = 0.812	r = 0.739	*r* = 0.889
				*p* < 0.001	*p* < 0.001	*p* < 0.001
**% Durnin-Womersley**				-	*r* = 0.705	*r* = 0.812
					*p* < 0.001	*p* < 0.001
**% Faulkner**					-	*r* = 0.916
						*p* < 0.001

BMI: Body mass index; %: percentage of fat mass.

**Table 6:** Percentile relationship of the sums of skinfolds and the kilograms and percentage calculated.

Percentile	BMI (kg/m^2^)	Sum of 6 Skinfolds (mm)	Sum of 8 Skinfolds (mm)	FM Kerr (kg)	FM Durnin-Womersley (kg)	FM Faulkner (kg)	FM Carter (kg)	% Kerr	% Durnin-Womersley	% Faulkner	% Carter
5	19.73	44.00	56.20	13.77	7.68	6.24	4.69	20.16	11.30	10.19	7.20
10	20.48	47.60	60.75	14.71	8.71	6.81	5.07	21.43	12.49	10.60	7.58
15	20.97	51.45	65.99	15.40	9.37	7.12	5.41	22.30	13.42	10.91	7.99
20	21.36	54.90	69.85	16.06	10.00	7.48	5.65	23.12	14.22	11.28	8.35
25	21.72	58.74	74.24	16.70	10.61	7.76	5.95	23.80	14.90	11.60	8.75
30	22.05	61.50	78.50	17.22	11.17	8.06	6.25	24.52	15.61	11.90	9.04
35	22.40	64.50	82.50	17.84	11.69	8.33	6.49	25.24	16.36	12.21	9.36
40	22.67	67.78	85.75	18.42	12.27	8.63	6.75	25.89	17.07	12.52	9.70
45	22.98	71.00	89.50	18.89	12.76	8.90	7.01	26.57	17.77	12.82	10.04
50	23.29	74.20	93.90	19.48	13.25	9.20	7.26	27.41	18.55	13.13	10.38
55	23.60	77.30	98.25	19.96	13.78	9.48	7.54	28.47	19.25	13.45	10.70
60	23.90	81.20	103.00	20.52	14.34	9.79	7.83	29.28	20.18	13.82	11.11
65	24.21	85.50	108.00	21.30	14.97	10.14	8.16	30.21	21.18	14.20	11.57
70	24.54	89.00	112.93	21.99	15.48	10.50	8.46	31.14	22.23	14.60	11.93
75	24.87	94.30	119.43	22.67	16.21	10.91	8.81	32.28	23.31	15.04	12.49
80	25.25	99.00	125.20	23.52	16.86	11.37	9.20	33.59	24.50	15.54	12.98
85	25.78	104.52	132.77	24.33	17.73	12.00	9.73	35.17	26.10	16.19	13.56
90	26.40	112.00	142.02	25.73	18.88	12.94	10.49	37.18	28.16	16.80	14.35
95	27.42	120.85	153.72	27.63	20.37	14.31	11.67	39.48	30.62	17.81	15.28

BMI: Body mass index; FM: fat mass weight; %: percentage of fat mass.
